# Willingness to migrate—a potential effect of burnout? A survey of Hungarian physicians

**DOI:** 10.1186/s12960-018-0303-y

**Published:** 2018-08-10

**Authors:** Zsuzsa Győrffy, Diana Dweik, Edmond Girasek

**Affiliations:** 1 Institute of Behavioural Sciences, Semmelweis University, Nagyvárad square 4, Budapest, H-1089 Hungary; 2 Department of Obstetrics and Gynecology, Healthcare Service Centre of Csongrád County, Kórház st. 2, Makó, H-6900 Hungary; 3 Health Services Management Training Centre, Semmelweis University, Kútvölgyi st 2, Budapest, H-1125 Hungary

**Keywords:** Burnout, Willingness to migrate, Physicians, Workload, Turnover

## Abstract

**Background:**

Burnout worldwide and migration of caregivers are among the most important challenges of the twenty-first century health care.

**Methods:**

Quantitative, online survey of Hungarian physicians (*n* = 4 784) was performed in 2013. A link to an anonymous, self-administered questionnaire was sent to all potential participants, namely to the registered members of the Hungarian Medical Chamber with a valid e-mail address. Linear regression analysis was used to determine the risk factors of burnout. The association between physicians’ burnout and their willingness to migrate was determined by binary logistic regression analysis.

**Results:**

Moderate/mild level of personal accomplishment was detected in 65% of respondents, whereas moderate/severe level of emotional exhaustion and depersonalization was detected in 49% and 46%, respectively. Single male physicians younger than 35 composed the cohort with the highest risk for developing burnout. Higher daily working hours and multiple workplaces contribute to the risk of developing burnout.

According to logistic regression analysis, the intention to work abroad was affected by the emotional exhaustion dimension of burnout (OR = 1.432) and depersonalization had a tendency to have an impact on the willingness to migrate.

**Conclusions:**

We assume that there is a circular causality between burnout and the willingness to migrate. Burnout increases the willingness to work abroad, whereas contemplating migration might evoke a certain degree of depersonalization in caregivers who are in a dilemma.

## Background

The worldwide healthcare workforce crisis is a permanent problem nowadays. The main factors of this phenomenon are the following: shortage of healthcare workers, aging and burnout of physicians and a higher demand for chronic care. The migration of healthcare workers has also been a highlighted issue in the past four decades. Since the 1970s, migration has become characteristic in all parts of the world (from developing countries to developed ones; from Western Europe to the United States of America; from Western European countries to other Western European countries) [1, 2]. Since the European Union accession in 2004, this issue has become more and more important as a consequence of the vanishing borders that have brought the Western European labour market closer to Hungarian employees [3, 4].

The EU predicts that over the next 50 years, the number of people over the age of 65 will double [5]. In parallel, the average age of the physicians is also rising. According to the EU forecast, around 60 000 doctors have to retire within 2 years (2020) in Europe [5]. In Hungary, according to the data of the National Health Service Center, 51% of the practicing physicians are older than 49 years, whereas the issue of migration affects mostly the young age group [6]. According to the Hungarian data, it is the 55–60-year-old age group that is the most populous of all the practicing cohorts of medical doctors, and serious shortage of labour is expected when they retire [6]. For example, the aging of general practitioners and the vacancy of their practice has been one of the main concerns of Hungarian health care for several years. While approximately 200 permanently vacant practices were registered in 2006 and 2007, this figure has become 303 by 2016. Furthermore, 40% of the GPs currently practicing in Hungary are older than 60 years of age [7]. In Hungary, the increasing number of participants in medical education could be a potential answer to this issue.

Approximately, 800–600 physicians have set up claim for the certification for working abroad annually between 2010 and 2015 and this number is showing a decreasing trend [8]. However, this number does not necessarily reflect the degree of real migration; rather, it can be a sign of certain physicians’ strong intention to work abroad [6]. In fact, just half or one third of these physicians have worked abroad [8]. Among those setting up claim for the official certificate, it is the rate of the 25–29-year-old trainee cohort that shows the most dynamic increase [8].

According to a previous Hungarian study, the most important motivation factors of one’s willingness to work abroad are salary, quality of life, working conditions and career options [9]. Both international and domestic research support that along with the growing emphasis on the above-mentioned factors, significant vocational alterations have been witnessed. These studies highlight that intrinsic motivations such as altruism are still crucial but not the only vocational factors [10]. Factors related to ‘controllable lifestyle’, such as salary, workplace, career options and opportunities of working abroad, are almost equally important compared with the helper motivation.

The issue of burnout is another considerable challenge of the twenty-first century health care. Burnout is the ‘state of mental and physical exhaustion caused by one’s professional life’ [11]. Maslach and Jackson created a multidimensional theory of burnout based on this definition. In this theory, three basic dimensions of burnout are explored: emotional exhaustion, depersonalization (negative attitude toward patients, colleagues and work) and the sense of decreased personal accomplishment, as the third factor [12]. Furthermore, according to Maslach et al., the harmony between the individual and his or her work and working conditions is among the key predictors of burnout. The six components of working conditions are the following: workload, sense of control, strain, workplace community, ‘fairness’ and harmony of the common values. The lack of these components is the most important moderating factor of burnout [13].

Based on the above-mentioned definitions, burnout is one of the most extensively investigated fields of research related to health care workers. Several studies highlight that burnout of caregivers is not a private issue, since it can have severe impact on the frequency of complications and malpractice [14–16]. According to the meta-analysis of Salyers et al., both patient safety and patient satisfaction are in close correlation with the extent of burnout [17]. Burnout decreases one’s efficacy and productivity and increases the number of days out of work, the possibility of leaving the profession and premature retirement [18, 19].

### Aims of the study

Several studies have associated the employees’ burnout with their leaving the profession [20, 21]. However, we could hardly find any studies on the potential link between burnout and one’s willingness to work abroad. According to our assumption, burnout of caregivers and their willingness to migrate are in potential correlation. In other words, the wish to change one’s work and life conditions can be a plausible response to emotional exhaustion, depersonalization and decreased personal accomplishment. The intention to work abroad could fulfil this wish. Our aim was to explore the potential association between the willingness to migrate and the degree of the different dimensions of burnout.

## Methods

A quantitative, online survey of physicians and dentists working in Hungary was conducted between 9 May and 15 July, 2013. The survey was performed with the permission of the Hungarian Medical Chamber. Ethical permission was obtained from the Ethical Committee of Semmelweis University, Budapest (No: 60/2013).

A link to an anonymous, self-administered questionnaire was sent to all potential participants, namely to the registered members of the Hungarian Medical Chamber with valid e-mail address (*n* = 42 342), which means 35 867 medical doctor and 6 475 dentists. This was followed by four reminder e-mails. Response rate was 13.24% (*n* = 5 607), breaking it down by profession, response rate was 12.71% among dentists (*n* = 823) and 13.34% among medical doctors (*n* = 4 784). Due to the special nature of their profession, dentists were examined as a separate group. In the present analysis, we limited our attention to medical doctors (*n* = 4 784). We considered a questionnaire complete in case the participant responded to at least 90% of the questions. All international studies emphasize that surveys of physicians have lower response rates compared with the general population even in case of traditional, paper-based surveys [22, 23]. The response rates of studies of physicians average about 10% lower than those of the general population. Studies on physician surveys have shown that lower response rates were the result of using web surveys alone compared with other survey designs [23].

Data were weighted by gender, age and type of profession (physicians vs. dentists), according to the characteristics of members of the Hungarian Medical Chamber. After three-dimensional weighting (gender, age and type of profession), the distribution of data concerning the region (counties) and type of workplace (general practice, in-patient and out-patient care) were compared with the same type of data from the Hungarian Central Statistical Offices [24]. As a result of the comparison, we found distributional differences neither by regions nor by type of workplace. Therefore, we considered our sample material representative.

### Measurements

During the analysis of socio-demographic data, we used a five-category variable for respondents’ age (24–35, 36–45, 46–55, 56–65 years old or older than 65). We gathered information on marital status and the number of children. In the present analysis, the type of primary workplace was a four-category variable (general practice, out-patient and in-patient care and other). The number of daily working hours, the amount of shift work and the number of workplaces were handled as continuous variables.

For the assessment of burnout, we used Maslach Burnout Inventory [25, 26]. MBI is a 22-item questionnaire which assesses burnout by three different subscales: emotional exhaustion (EE), depersonalization (DP) and personal accomplishment subscales (PA). A seven-point Likert scale (from zero to six) was used to assess the frequency of certain work-related emotions. The 22 items put up three subscales (EE, DP and PA). EE consists of nine statements and assesses the feelings of being overextended and exhausted in one’s workplace (e.g. ‘I feel depressed at work’). DP consists of five items and measures an unfeeling and impersonal response toward recipients of one’s service, care treatment or instruction (e.g. ‘I don’t really care what happens to some recipient’”). PA consists of eight statements that assess feelings of competence and success in one’s work (e.g. ‘I have accomplished many worthwhile things in this job’) [25].

According to the scores reached on each subscale, severity of burnout is assessed as mild, moderate and severe. Severe burnout among physicians is defined as EE > 27; DP > 10; PA < 33 [25]. The Cronbach alpha of EE, DP and PA in our study was 0.909, 0.767 and 0.818, respectively.

The willingness to migrate was assessed by the following statement: ‘Do you plan to work abroad in the following one or two years?’ (yes or no). If the answer was yes, the following question was whether the respondent wished to work abroad as a medical doctor or not.

We also asked if the respondent had already taken any preparative measures in order to work abroad. Response options were the following: ‘Yes, I have written contract with a foreign institution’; ‘Yes, I have oral contract with a foreign institution’; ‘Yes, I am in contact with a foreign institution (via mail or phone)’; ‘I do not have a contract yet’; ‘I have already inquired about the working options’; ‘It is among my future plans’.

### Statistical analysis

Descriptive statistics were used to determine the prevalence of burnout among Hungarian physicians. Linear regression model was used to determine the risk factors of the dimensions of burnout. In the analysis, we examined the impact of several socio-demographic and work-related variables on all three dimensions of burnout, such as gender, age, number of workplaces, number of daily working hours, doing shift work, marital status and type of primary workplace.

After the explanation model of the dimensions of burnout, binary logistic regression model was executed for the two-category variable of the willingness to migrate. In binary logistic regression analysis, we used binary variables of EE, DP as dependent variables, control variables were gender, marital status (single/living with partner) and doing shift work (yes/no) were used as two-category variables, whereas age, number of workplaces, number of daily working hours were continuous variables. We created four-category variables from the variable ‘type of primary workplace’ (general practice, outpatient department, inpatient department and others).

A threshold of *p* < 0.05 was established to consider an association significant. Statistical analyses were conducted using the SPSS 21.0 for Windows.

## Results

### Socio-demographic data

There were 2 269 male (47.4%) and 2 515 female (52.6%) physicians in the study. Mean age of respondents was 52.5 years, the minimum age was 24 years old and the maximum was 84. (The median age was 52 years.) The age distribution of the sample shows that the two age groups with the largest headcount were those in the 46–55 and the 56–65-year-old groups, which formed 10.8% and 11.9% of the respondents respectively.

More than two thirds of respondents (69.2%) were married, 12.1% were cohabiting, 7.5% were divorced, 7% were single and 4.3% were widowed.

Two fifth (41%) of the respondents had two children. Childless was 18.5% and 18.5% had one child, while 16.1% and 4.2% had three and four children, respectively. The mean number of children was 1.7.

Almost half of the practicing physicians (48.1%) had specialization in any field of medicine, 11% were trainees, whereas 16.4% worked as pensioners. Entrepreneur physicians were 18.4%, 2.2% were university teachers and 0.4% were researchers. Only 1.5% was on maternity leave. With regard type of workplace, 42.3% and 17.6% worked in in-patient and out-patient care, respectively. One quarter were GPs (25.5%), and 14.6% worked somewhere else (civilian organization, civil service or private health care). Almost half (48.6%) of the participants had just one workplace. More than one quarter (25.6%) and 11.8% had two or three workplaces, respectively, 5 % had four or more workplaces simultaneously and 9.1% of the respondents reported having no job at all. The mean number of workplaces was 1.58.

The mean number of specializations was 1.4. Two fifths (40%) of the sample had one, one third had two and almost 11% had three different specializations, while 13.8% had no specialization. Information was gathered about the type of the specialization that was first acquired and about the primarily used type (in case of more than one specialization) (Table 1).

**Table 1:** Specialization

Specialization	Number	Percent
Addictology	256	6.0
Jawbone, oral, maxillar and mandibular bones surgery	5	0.1
Internal medicine	849	19.7
Dermatology	74	1.7
Paediatrics	535	12.4
Physical medicine and rehabilitic medicine	16	0.4
Occupational medicine	103	2.4
Otolaryngology	98	2.3
Gastroenterology	1	0.0
Geriatrics	2	0.1
Paediatric and youth psychology	9	0.2
Paediatric dentistry	3	0.1
Paediatric surgery	3	0.1
Family Medicine (general practice)	484	11.2
Defence and catastrophe medicine	2	0.0
Vasculosurgery	5	0.1
Forensic medicine	16	0.4
Infectology	18	0.4
Cardiology	9	0.2
Endodontic and prosthetic dentistry	5	0.1
Preventive and public medicine	50	1.2
Neurology	143	3.3
Nuclear medicine (isotopdiagnostic)	14	0.3
Orthopaedics-traumatology	94	2.2
Medical laboratory diagnostics	114	2.6
Medical microbiology	17	0.4
Oxyology (ambulance medicine)	47	1.1
Patology	98	2.3
Psychiatry	187	4.3
Radiology	201	4.7
Aviation medicine	5	0.1
Rheumatology	79	1.8
Surgery	283	6.6
Parodontology	3	0.1
Ophtalmology	117	2.7
Cardiosurgery	2	0.1
Obstetrics and gynaecology	166	3.8
Transfusiology	10	0.2
Pulmonolgy	126	2.9
Urology	55	1.3
All	4 306	100.0

### Willingness to migrate

Most of the respondents (82%) did not plan to work abroad in the forthcoming one or 2 years, whereas 16.6% and 1.4% planned to work abroad either as physician or not as physician, respectively.

Among those who plan to work abroad, 17.1% had written or oral contract, and 50% were actively searching the options of working abroad (without any contract yet). There were 32.9% who had plans of migration in the near future but had not taken any measures to contact a foreign health care institution.

### Burnout of physicians

The mean scores of EE, DP and PA were 19.44 (SD = 12.8), 6.13 (SD = 6.1) and 34.42 (SD = 9.2), respectively. Mild, moderate and severe levels of emotional exhaustion were detected in 49.4%, 27.1% and 22.2% of the respondents, respectively. Mild, moderate and severe levels of depersonalization were detected in 57.3%, 23.7% and 19% of the respondents, respectively. Mild, moderate and severe decrease of personal accomplishment was detected in 34.2%, 26.1% and 39.7% of the respondents, respectively (Table 2).

**Table 2:** MBI subscales in the physician sample

	Emotional exhaustion % (*n* = 4 514) and cumulative percent	Depersonalization % (*n* = 4 477) and cumulative percent	Personal accomplishment % (*n* = 4 487)
Mild	49.4 (2229) 49.4	57.3 (2566) 57.3	34.2 (1534) 34.2
Moderate	27.1 (1225) 76.5	23.7 (1062)	26.1 (1172) 60.3
Severe	22.2 (1060) 100	19 (849) 100	39.7 (1781) 100

### Risk factors of burnout

Six variables had significant effect on emotional exhaustion (*R*^2^ = 0.119 for the total model): number of daily working hours, age, number of workplaces and type of primary workplace (in/out-patient department/GP/others). Number of daily working hours had the strongest effect, which was followed by the age of the respondent and inpatient practice and general practice as primary workplace (Table 3).

**Table 3:** Linear regression analytic model of burnout

Explanatory variables of EE	Adjusted beta	Explanatory variables of DP	Adjusted beta	Explanatory variables of PA	Adjusted beta
Constant		Constant		Constant	
Age	− .168	Age	− .299***	Age	NS
Gender	NS	Gender	− .142***	Gender	NS
Shift work	NS	Shift work	0.76***	Shift work	.076*
In-patient practice	.097***	In-patient practice	NS	Type of workplace	NS
Outpatient practice	NS	Outpatient practice	NS	Outpatient practice	.051*
GP	.064**	GP	NS	GP	.078*
Other	−.038***	Other	0.53**	Other	NS
Number of workplaces	0.53**	Number of workplaces	NS	Number of workplaces	.081**
Marital status	NS	Marital status	−.049*	Marital status	.074***
Daily working hours	.206***	Daily working hours	−.047*	Daily working hours	.051*
*p* value for the full model	*p* < 0.001	*p* value for the full model	*p* < 0.001	*p* value for the full model	*p* < 0.001
Adjusted *R*^2^	0.119	Adjusted *R*^2^	0.134	Adjusted *R*^2^	0.034

*NS* not significant

*p < 0,05, **p < 0,01,***p < 0,001

In case of depersonalization, six significant variables entered the model (*R*^2^ = 0.134). Age had the strongest (negative) effect, followed by gender (women were at smaller risk to be affected by depersonalization), doing shift work, working primarily at a private service provider (negative correlation) and marital status (lower DP scores for those who lived in a partner relationship (Table 3).

The effect of five variables proved to be significant in case of decreased personal accomplishment (*R*^2^ = 0.034). Number of workplaces, doing shift work, marital status and working as a GP had the strongest effect (Table 3). Personal accomplishment decreased in line with increasing number of workplaces, doing shift work and working as a GP (Table 3).

### Risk factors of the intention to work abroad

Primary aim of our study was to examine the effect of the three dimensions of burnout on the decision to work abroad. In the first step, we examined the correlation between EE, DP, PA and the willingness to work abroad. In case of EE and DP, there was a significant correlation (*p* < 0.000 and *p* < 0.004, respectively). In case of PA, the correlation was not significant (*p* < 0.204). In the next step, we built a logistic linear model to unveil the association between EE and DP and the willingness to migrate. In binary logistic regression analysis, binary variable of willingness to migrate was used as dependent variable, while independent variables were gender, age, type of workplace, daily working hours, doing shift work and marital status. After controlling to these variables, EE, beside other traditional risk factors, turned out to be an important explanatory factor. The result of the analysis showed that depersonalization had tendency-like effect on the willingness to migrate (Table 4*).*

**Table 4:** The association between moderate and high EE and DP and the willingness to migrate

Dependent variables	Unadjusted OR (95% CI)	Adjusted OR (95% CI)*
EE	1.581**** (1.431–1.732)	1.432**** (1.216-2.150)
DP	1.403** (1.281–2.110)	1.016***** (0.957–1.248)

*Adjusted for age, gender, number of workplaces, daily working hours, shift work and marital status

**p < 0.05

***p < 0.01

****p < 0.001

*****NS not significant

## Discussion

We investigated work-related risk factors of burnout and the association between burnout and one’s willingness to migrate in our nationwide, representative survey of Hungarian physicians. We found that decreased personal accomplishment was the most prominent of all three dimensions of burnout among them. Moderate or severe level was reached by 65.1% of the respondents on PA. This was followed by EE, where moderate and severe level was reached by 49%. On the DP, moderate or high level was reached by approximately 46%. Heavy workload (more than 40 working hours a week and having more than one workplace), young age and doing shift work correlated significantly with EE, which is the core dimension of burnout. These results are in line with the international trends: heavy workload (daily working hours, doing shift work) and young age were associated with high scores of all dimensions of burnout [27, 28].

Young physicians’ cohort (in our study, single males under 35 years) is at extreme risk of burnout. However, previous studies found rather conflicting results concerning the correlation between age, burnout and working experience of physicians. There are studies that showed that the youngest, entrant age group was mostly affected by burnout. According to their results, heavy physical and mental burden, the weight of responsibility along with the lack of control over one’s work, sleep deprivation and decreasing recreation time resulted in severe mental vulnerability of young physicians [29–31]. The authors of a Belgian study found significantly elevated cortisol levels in residents, which was interpreted as an obvious sign of high stress response [32]. Although a certain level of stress on young physicians helps effective and motivated coping, chronic stress burden leads to decreasing motivation and non-adaptive coping mechanisms, such as suicidal ideation, psychosomatic disorders, etc. [33, 34]. This is in line with the findings of another study: high level of perceived stress was reported by more than 50% of young doctors [35]. In contrast, Dyrbye et al. found in one of their latest studies that physicians having worked for 11–20 years were the least satisfied with their chosen specialization, and they had the highest scores generally on burnout scales, especially on EE [36].

Our multivariate analysis showed that heavy workload and having more than one workplace indirectly increase the risk of burnout, whereas emotional exhaustion and depersonalization dimensions of burnout have direct impact on the willingness to migrate. The World Health Organization in its 2008 analysis found that the following factors have the most severe impact on health care workers’ migration and turnover from work: heavy or dangerous working conditions, insufficient resources, economic instability and the lack of career options [37]. The report stressed ‘heavy working conditions’ which seem to be key factors in the process of burnout [38]. However, our analysis highlighted that the key dimension of burnout (emotional exhaustion) had an obvious impact on the willingness to migrate. This is in line with the conclusions of the meta-analysis of Lee et al.: there is a strong, positive correlation between burnout and migration [39]. Pantenburg et al. also found in their study of German physicians that severe emotional exhaustion and depersonalization increased the odds of working abroad [40]. Previous studies showed that dissatisfaction with one’s job increased the risk of turnover from the health care sector [41]. A meta-analysis from 2014 found that burnout was in correlation with the length of sick leave and the possibility of turnover from the present workplace [19]. Ruitemburg et al. reported that physicians having reached high scores on EE and DP perceived their own personal accomplishment significantly lower [42].

The unique approach of our study is the explicit association between burnout and the willingness to migrate, an issue investigated by only a handful of studies. The relatively low response rate is a limitation; however, multi-dimensional weighting was aimed to create respondent groups that were more representative. A further limitation is that we used a single-item tool for the assessment of the willingness to migrate. According to our latest survey, the willingness to work abroad is decreasing. (2013, 16.7% vs. 2017, 10.2%) [43].

## Conclusion

Studies on malpractice cases, hospital infections and migration highlighted that prevention of burnout was the most cost-effective tool of health policy. Prevention of burnout has proved to be feasible both on organizational or individual level [44–46]; therefore, it could play an enormous role in the effectiveness of the health care system. Results of our study showed that burnout can be seen as a consequence of one’s dissatisfaction with their working conditions. Nevertheless, emotional exhaustion equals not only dissatisfaction with one’s working conditions but also a severe emotional burden that derives from a caregiver’s work in general, which suggests that this is a quite complex phenomenon. The number of physicians who take on a job abroad is much less than those who plan to, in reality. This fact might confirm the assumption that the planning to work abroad is a possible coping strategy against burnout. Idealising another workplace can be a way to handle the matter of emotional exhaustion.

We can also assume that there is a circular causality between burnout and the willingness to migrate. Burnout increases the willingness to work abroad, whereas contemplating migration might evoke a certain degree of depersonalization in caregivers who are in a dilemma.

## Abbreviations

DP: Depersonalization subscale
EE: Emotional exhaustion subscale
GP: General practice
MBI: Maslach Burnout Inventory
OR: Odds ratio
PA: Personal accomplishment subscale
SD: Standard deviation
